# Transcription Factor IRF4 Dysfunction Affects the Immunosuppressive Function of Treg Cells in Patients with Primary Immune Thrombocytopenia

**DOI:** 10.1155/2019/1050285

**Published:** 2019-07-10

**Authors:** Meiwen Tang, Luya Cheng, Feng Li, Boting Wu, Pu Chen, Yanxia Zhan, Fanli Hua, Zhihui Min, Yang Ke, Chanjuan Liu, Ling Yuan, Lihua Sun, Hao Chen, Lili Ji, Yunfeng Cheng

**Affiliations:** ^1^Department of Hematology, Zhongshan Hospital Fudan University, Shanghai 200032, China; ^2^Department of Hematology, Zhongshan Hospital Qinpu Branch, Fudan University, Shanghai 201700, China; ^3^Department of Transfusion Medicine, Zhongshan Hospital Fudan University, Shanghai 200032, China; ^4^Department of Clinical Laboratory, Zhongshan Hospital Fudan University, Shanghai 200032, China; ^5^Institute of Clinical Science, Zhongshan Hospital Fudan University, Shanghai 200032, China; ^6^Department of Thoracic Surgery, Zhongshan Hospital Xuhui Branch, Fudan University, Shanghai 200031, China; ^7^Center for Tumor Diagnosis & Therapy, Jinshan Hospital, Fudan University, Shanghai 201508, China

## Abstract

**Background:**

Th17/Treg balance skews towards Th17 in ITP patient. IRF4 has been highlighted for its close relationship to the immunosuppressive function of Treg cells and the IL-17 synthesis in CD4^+^ T cells. This study was aimed at examining the effects of IRF4 to the Th17/Treg cells in patients with ITP.

**Methods:**

Treg and Teff cells were isolated from PBMCs of newly diagnosed ITP patients. The percentages of CD4^+^CD25^hi^Foxp3^+^Treg cells and the CD3^+^CD4^+^IL-17^+^Th17 cells were detected by flow cytometry. After being cultured, the supernatants of Tregs were collected for IL-10 concentration test. The IRF4 levels of Tregs were measured. Teffs were cultured alone or with Tregs for 24 hours. Then the supernatants were collected for IL-17 concentration test. The binding intensity of IRF4 to the gene IL-10 in Treg cells was detected by ChIP-qPCR. Metabolic assays for Teffs and Tregs were performed with Agilent Seahorse XF96 Analyzer.

**Results:**

The secretion of IL-10 by Tregs was decreased in ITP patients. The intensity of IRF4 binding to IL-10 DNA of Tregs in patients was higher than that of normal controls and Teffs in ITP patients. The expressions of IRF4 of Tregs in ITP patients were remarkably lower than that of healthy controls. The percentage of Th17 cells in healthy controls was significantly increased after IRF4 mRNA silencing. Abnormal metabolism of Treg and Teff cells was found in ITP patients.

**Conclusion:**

The skewed ratio of Th17/Treg cells and dysfunction of Treg cells in newly diagnosed ITP patients was at least partly caused by IRF4 dysfunction. The underlying mechanism might be the impact of IRF4 on the metabolism of Treg and Teff cells.

## 1. Introduction

Primary immune thrombocytopenia (ITP) is an autoimmune heterogeneous disorder presenting with decreased platelet count and increased bleeding risk. Both impaired platelet production and increased platelet destruction are significant in the pathogenesis of ITP, in which autoreactive T cells and innate immune system play important roles [1, 2].

CD4^+^CD25^hi^Foxp3^+^Treg cells and CD3^+^CD4^+^IL-17-producing Th17 cells are two subsets of CD4^+^ T helper (Th) cells [2]. TGF-*β* and IL-10 producing Treg cells are crucial immune response regulators in autoimmune diseases [3]. It is known that decreased number and dysfunction of Treg cells play important role in ITP [4]. IL-17 produced by Th17 cells lead to subsequent inflammation factors release and tissue damage in ITP and other autoimmune disease [5, 6]. Th17/Treg balance is regarded as a key factor in immune homeostasis; a variety of autoimmune diseases were caused when Th17/Treg balance is skewed [7–9]. The ratio of Th17/Treg cells in active SLE patients is significantly higher than that in inactive patients and healthy controls, which associate with the severity of disease [10]. Our previous study indicated that the percentage of Treg cells in ITP patients was significantly lower than that of healthy controls, and the ratio of Th17/Treg correlated with the disease activity of ITP [11].

The transcription factor interferon regulatory factor (IRF4) has been known to be associated with immune regulation and is essential to the differentiation of the effector CD4^+^ T helper cell subsets [12–17]. The previous study in mouse found that the upregulation of IRF4 is dependent on the expression of Foxp3 [18]. In patients with autoimmune diseases, abnormality of Foxp3 expression resulted in IRF4-deficiency, which caused incapable of starting the transcription of downstream gene and impaired immunosuppressive function of Treg cells [18]. IRF4 is a critical transcription factor both for Treg and Th17 cells in CD4^+^ T cells [19].

Interleukin-10 (IL-10) is an important regulatory cytokine of Tregs in inflammatory conditions [20]. IL-10 elevates Tregs' suppression against Teffs, while Tregs of ITP patients could not effectively produce enough IL-10 to sufficiently inhibit Teffs [21, 22]. Effective corticosteroids treatment improved the IL-10 production of Tregs in ITP patients, which suggested that IL-10 levels might associate with ITP disease states. IL-10-producing Tregs directly inhibit Th17 and IFN-*γ*^+^IL-17^+^double-producing T cells (IFN-*γ*^+^IL-17^+^Th cells) via IL-10 secretion [23–27]. Recent studies showed that the increase of IL-10-producing Foxp3^+^Tregs was accompanied with disease remission in several different systematic autoimmune diseases, such as experimental autoimmune encephalomyelitis, diabetes, and arthritis [28–30].

Via metabolic reprogramming, CD4^+^ effector T cells and Treg cells support their proliferation and immunological function [31]. Induced Treg cells have been shown to utilize mainly a distinct metabolic program based on mitochondrial oxidation of lipid and pyruvate [32–34]. CD4^+^CD25^−^Teff cells are dependent on aerobic glycolysis for proliferation and inflammatory functions [35, 36]. Whether there is any correlation between the dysfunction of Treg cells and the cell metabolism status remains to be explored.

One the other hand, previous study has shown the impaired ability of IL-10 secretion of Treg cells, which suggests the impairment of Treg cells regulatory function in ITP patients [22]. IL-10 is IRF4-dependent downstream cytokine of Treg cells [18, 37]. It is unclear if there are any associations between the IRF4 with the Th17/Treg imbalances and abnormal secretion of IL-10 in ITP patients, or how the cell metabolism involved in the disease progression is. In the current study, we aimed to investigate the expressions of IL-10 and the immunosuppressive function of Treg regulated by IRF4 and the impact on the balance of Th17/Treg in newly diagnosed ITP patients, in order to better understand the pathogenesis of ITP, and could pave ways to specific immune therapy targeting ITP and other autoimmune diseases.

## 2. Materials and Methods

### 2.1. Patients and Healthy Controls

Thirty-six patients with newly diagnosed ITP were enrolled into this study (20 females and 16 males, age range 19-77 years, and median age 49 years) (Supplementary Table 2). ITP was diagnosed per criteria proposed by an international working group [1, 38, 39]. Twenty healthy volunteers were recruited as normal controls (normal control group, NC group) (Supplementary Table 3). The study was approved by the institutional review board of Zhongshan Hospital, Fudan University. Written informed consent was obtained from each patient prior to the enrollment.

#### 2.1.1. Sample Preparation [22]

Twenty milliliter peripheral venous blood samples of each study subject were collected in ethylenediaminetetraacetic acid-treated tubes and diluted 1:2 with Hanks balanced salt solution (HBSS) before Ficoll-Hypaque gradient centrifugation (2, 200 rpm at room temperature for 15 min). Washed and resuspended isolated peripheral blood mononuclear cells (PBMCs) were cryopreserved in fetal bovine serum containing 10% dimethyl sulfoxide (DMSO) and stored in liquid nitrogen for future studies.

#### 2.1.2. Flow Cytometry Analysis [22]

To test CD4^+^CD25^hi^Foxp3^+^Treg cells, 1 × 10^6^ PBMCs were stained with CD4 FITC (eBioscience, San Diego, California, USA, Cat# 11-0048-42) and CD25 PE-CY7 (BD Bioscience, Cat# 506225). To detect Th17 cells, 1 × 10^6^ PBMCs were adjusted concentration as 5 × 10^5^/ml in RPMI1640 medium supplemented with 10% heat-inactivated fetal bovine serum, 2 mM L-glutamine, 200 U/ml penicillin, and 100 *μ*g/ml streptomycin and cultured in 24-well plates overnight. Before stimulation, the supernatants were collected for IL-17 ELISA test. The concentration of PBMCs were stimulated with 50 ng/ml phorbol myristate acetate (PMA, Sigma-Aldrich, St. Louis, Missouri, USA, Cat# P8139) and 500 ng/ml ionomycin (Sigma-Aldrich, Cat# I9657) for 4 hours. End in 2 hours after incubation, 1 ul/ml brefeldin A solution (BFA, Biolegend, Cat# 420602) was added to the culture system. Then PBMCs were stained with CD4 FITC and CD25 PE-CY7. After the fixation and permeabilization step described above, the cells were stained with IL-17A PerCP-Cy5.5 (BD Bioscience, Cat# 560799). All steps are according to the manufacturer's protocol (Supplementary 4). Acquisition was performed on a FACS Aria II flow cytometer (BD biosciences, USA) and then analyzed using Flowjo software version 7.6.1 (Tritar Inc., San Carlos, California, USA).

#### 2.1.3. Cell Purification and Culture [22]

Dead cells were removed by dead cell removal kit (Miltenyi Biotec, Auburn, California, USA, Cat# 130-090-101). CD4^+^CD25^hi^Foxp3^+^Treg cells and CD4^+^CD25^−^Teffs were isolated from PBMC using the CD4^+^CD25^+^Treg cells isolation kit (Miltenyi Biotec, Cat# 130-091-301) according to manufacturer's instruction (Supplementary 4). The purification of Tregs and Teffs were determined by FACS Aria II flow cytometer (BD biosciences, San Jose, California, USA) using CD4 APC (Biolegend, San Diego, California, USA, Cat# 300514) and CD25 PE (Biolegend, Cat# 302605).

Before culture, 24-well round-bottom plates were preincubated with anti-CD3 (10 *μ*g/ml; Biolegend, Cat# 300414) at 37°C for 2 hours. Teffs were cultured with Tregs at 8:1 ratio or without Tregs. The cells were cultured in 1 ml per well in 24-well round-bottom plates which were preincubated and stimulated with anti-CD28 (2.5 *μ*g/ml, Biolegend, Cat# 302914) and 10 ng/ml interleukin-2 (IL-2; Biolegend, Cat# 589102).

#### 2.1.4. RNA Silencing

The siRNAs for IRF4 and Negative control siRNA were purchased from GenePharma. RNA strand sequences were sense: 5'- GGCUUGGGCACUGUUUAAATT-3' and antisense: 5'-UUUAAACAGUGCCCAAGCCTT (IRF4-Homo-342); sense: 5'-GCGCUUUGAACAAGAGCAATT-3' and antisense: 5'-UUGCUCUUGUUCAAAGCGCTT (IRF4-Homo-422). CD4^+^CD25^hi^Foxp3^+^Treg cells and Teffs were isolated from PBMCs of healthy controls and cultured for 24 h. The day before transfection, take 5×10^4^ CD4^+^CD25^hi^Foxp3^+^Treg cells inoculated on 24-well round-bottom plates. Take 20 pmol siRNA and 50 *μ*l DMEM into solution. Lipofectamin TM 2000 (Invitrogen) was diluted 1:50 in DMEM. siRNA/Lipofectamine compound formed after dilution and was added to the culture system, then it was incubated in the CO_2_ incubator (at the temperature of 37°C). After 6 hours, compounds were removed and cells were washed and then cocultured with 8 times of the number of CD4^+^CD25^−^Teffs for 24 hours. The cells were harvested for flow cytometry analysis of Th17 cells and the centrifugal supernatant was used for ELISA test for IL-17 concentration.

#### 2.1.5. ELISA

Supernatants from peripheral venous blood samples of patients and volunteers and the cell cultures were collected and stored at -80°C until the cytokine measurements. The secretions of IL-17A (R&D Systems, Inc., Cat# D1700) by coculture systems and IL-10 (R&D Systems, Inc., Cat# EL217B) by Tregs were measured in duplicate by ELISA following the manufacturer's recommendation. Results were expressed for each subject as cytokine concentration in pictogram per milliliter.

#### 2.1.6. Western Blotting

1 x SDS buffer (Cell Signaling Technology, Cat# 7722) was added to sorting CD4^+^CD25^hi^Foxp3^+^Treg cells from PBMCs. After cracking, samples were ultrasonically processed for 10 to 15 seconds. 20 *μ*l sample aliquots were then heated at 95°C for 5 minutes, and cooled on ice. After centrifuged for 5 minutes, sample aliquots were added on SDS-page gel (Cell Signaling Technology) and combined with biotin labeled protein molecular weight standard (Cell Signaling Technology, Cat# 7727) 10 *μ*l per lane. After electrophoresis of protein power transferred to nitrocellulose membrane (Cell Signaling Technology, Cat# 12369), nitrocellulose membrane was cleaned twice at room temperature by 25 ml TBS for 5 minutes each time, incubated in 25 ml sealing fluid at room temperature for 1 hour, and cleaned with 15 ml TBS/T 3 times for 5 minutes each time. After incubated primary antibodies, anti-IRF4 Ab (Cell Signaling Technology, Cat# 4964s) and *β*-actin (Cell Signaling Technology) were added. The protein bands were detected with an ECL Detection System (Santa Cruz Biotechnology. SignalFire™ ECL Reagent, Cell Signaling Technology, Cat# 6883).

#### 2.1.7. Real-Time PCR

Total RNA was isolated from Treg cells and reverse-transcribed to cDNA using an iSCRIPT cDNA synthesis kit (Bio-Rad). Real-Time PCR (qRT-PCR) was performed using an Applied Biosystems ABI 7500. The primers were purchased from Applied Biosystems and the primer sequences listed in Supplementary Table 1. Each sample was analyzed in triplicate. Relative gene expression was expressed upon normalization against 18S RNA.

#### 2.1.8. Chromatin Immunoprecipitation (ChIP)

CD4^+^CD25^+^Treg cells and CD4^+^CD25^−^Teffs were separated from PBMCs of ITP patients and healthy volunteers with magnetic bead and transferred to the centrifuge. Formaldehyde (270 *μ*l, 37%) was added and their final concentration was adjusted 1% for 10 min incubation at room temperature. Then 125 mM ice-cold glycine (505 ul 2.5 M) was added for another 5 min at room temperature. Cells were washed and supernatant was removed. Protease inhibitors were added to lysis buffer 1 and lysis buffer 2. Pellets were resuspended in 1mL lysis buffer 1 and swirled after 10 min at 4°C, then centrifuged and abandoned the supernatant. The pellets were resuspended in 300 *μ*L of lysis buffer 2 and placed on the ice for 30 min. Then the pellets sonicated on the ice with a Bioruptor. The generated fragments were approximately 500 bp long, as determined experimentally by Agileng 2100 software. 750 *μ*l IP buffer, 100 *μ*l 5% BSA, magnetic beads-antibody complex, and 4 *μ*g anti-IRF4 Ab (Cell Signaling Technology, Cat# 4964s) 30 *μ*l were added; 0.6 *μ*l Salmon Sperm DNA, 100 *μ*l fragments of chromosome, 10 *μ*l protease inhibitors, and PMSF 10 *μ*l were mixed well. After incubation at 4°C overnight, the beads were subsequently washed with buffers A, B, and C (according to the instructions), at 4°C with permanent rotation for 10 mins. Following two washes with TE buffer, samples were eluted in 150 *μ*l Chip Elution Buffer for 1 hour at 65°C. Samples were then added 8 *μ*l proteinase K and 6 *μ*l NaCI (5 M) and cross-links reversed at 65°C overnight. DNA was purified using QIAquick PCR Purification Kit (QIAGEN).

#### 2.1.9. Metabolic Assays of Treg and Teff Cells

Two hundred mL of Seahorse Bioscience calibrant (pH 7.4) was added to each well of a Seahorse Bioscience 96-well utility plate before the day of assay. The XF96 cell culture plate (Seahorse Bioscience) was coated with 10 *μ*L of Cell-Tak (BD Biosciences) which were prepared a working solution in sterile H_2_O. NaHCO_3_ 0.1 M (pH 8.0) 40 *μ*L was added to per well to neutralize and promote adsorption of Cell-Tak to the plate, incubated overnight at 37°C. Oligomycin, FCCP, and 2-DG (Sigma) were diluted and added as Agilent Seahorse XFe96 Extracellular Flux Assay Kit. OCR and ECAR were measured by XF96 extracellular flux analyzer (Seahorse Bioscience) as described [40]. OCR and ECAR values were normalized to cell number. Glycolytic capacity is distinguished by the difference between the ECAR following the injection of oligomycin and ECAR following glucose injection.

#### 2.1.10. Statistical Analysis

All analyses were performed using SPSS software (version 13.0; SPSS Inc., Chicago, IL, USA). Data are expressed as the mean ± SD. Normality was assessed by Shapiro-Wilk test. Student* t* test and Wilcoxon rank-sum (Mann-Whitney) test were used for data fulfilled normal distribution and for those did not, respectively. One-way analysis of variance or Kruskal Wallis testing was used for normal or nonnormal data, respectively. The least significant difference test was used for post hoc multiple comparisons. Two-sided* p *values <0.05 were considered statistically significant.

## 3. Results

### 3.1. The Ratio of Th17/Treg Cells Increased, While the Secretion of IL-10 Decreased in ITP Patients

**Figure 1:**
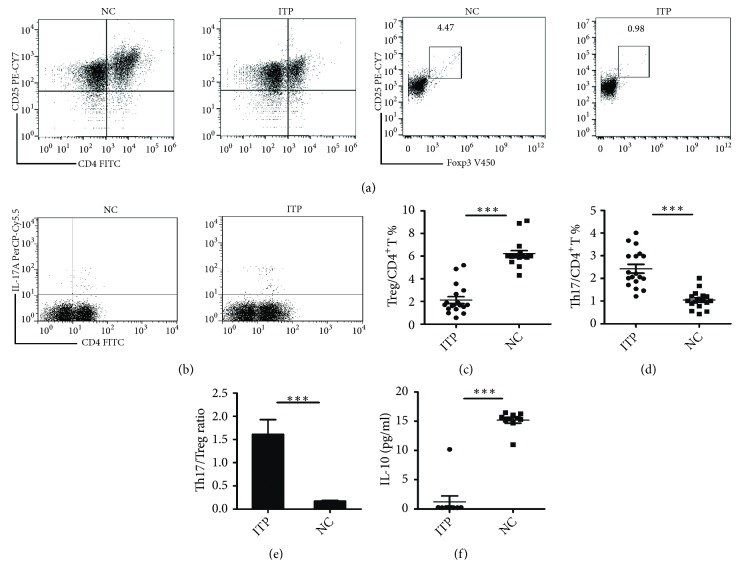
*The ratio of Th17/Treg cells increased; the secretion of IL-10 of Tregs decreased in ITP patients.* (a) Representative dot plots of Tregs (CD4^+^CD25^hi^Foxp3^+^Treg cells) in ITP and NC groups. (b) Representative dot plots of Th17 cells (CD4^+^ IL-17^+^ cells) in ITP and NC groups. (c) The percentage of Treg cells in CD4^+^ T cells of ITP and NC groups. (d) The percentage of Th17 cells in CD4^+^T cells of ITP and NC groups. (e) The ratio of Th17/Treg in ITP and NC groups. (f) The expression of IL-10mRNA of Tregs in ITP and NC groups.* NC: *normal control;* ITP:* ITP group; *∗p* < 0. 05; *∗∗p* < 0. 01; *∗∗∗p* < 0. 001.

The population of CD4^+^CD25^hi^Foxp3^+^ T cells from PBMCs of ITP patients and healthy volunteers were identified as Tregs. The percentage of Treg cells in ITP group was decreased, compared with that in control group ((2.12 ± 0.30)%* vs *(6.22 ± 0.27)%,* p*<0.001), Figures 1(a) and 1(c). The population of CD3^+^CD4^+^IL-17^+^ T cells was identified as Th17 cells, and their percentage was significantly greater than that in normal controls ((2.43 ± 0.12) %* vs* (1.05 ± 0.09) %,* p*<0.001), Figures 1(b) and 1(d). The ratio of Th17 cells to Treg cells increased in ITP patients when compared with NC group (1.61 ± 0.32* vs* 0.17 ± 0.02,* p*<0.001), Figure 1(e). The secretion of IL-10 by Tregs decreased in ITP group, compared with NC group ((1.23 ± 0.10) %* vs* (15.17 ± 0.49) %,* p*<0.001), Figure 1(f).

### 3.2. Abnormal Expression of Interleukin Regulatory Factor 4 Gene in Treg Cells of ITP Patients

**Figure 2:**
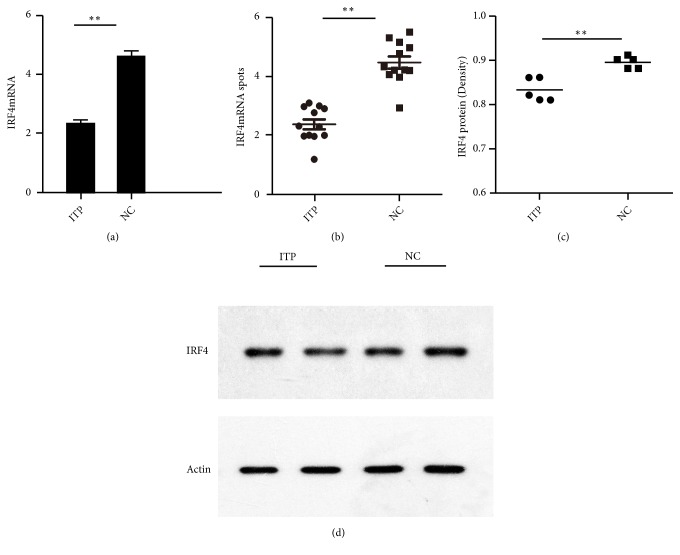
*Abnormal expression of IRF4 gene in Treg cells in ITP patients.* (a) The histogram of the expression of IRF4mRNA of Tregs in NC and ITP patients. (b) The scatter diagram of the expression of IRF4mRNA of Tregs. (c) The relative IRF4 protein of Tregs of NC and ITP groups by western blot. (d) The expression of IRF4 protein of Tregs in NC and ITP groups by western blot. *∗p* < 0. 05; *∗∗p* < 0. 01 *∗p*<0.05; *∗∗p*<0.01.

IRF4mRNA expression was evaluated in CD4^+^CD25^hi^Foxp3^+^Treg cells in 5 patients and 5 control subjects of NC groups. The expression of IRF4mRNA of Treg cells from ITP patients was significantly lower than that of NC group (2.17 ± 0.31* vs *4.40 ± 0.48,* p*=0.0077), Figures 2(a) and 2(b). The expression of IRF4 protein in Tregs of patients with ITP was identified with western blot. Densitometry analysis showed a significantly decreased of IRF4 protein level in patients with ITP, compared with NC group (0.83 ± 0.01* vs *0.91 ± 0.01,* p*=0.0014), Figures 2(c) and 2(d).

### 3.3. The Target Gene IL-10 of IRF4 Was Associated with the Inhibition Function of Treg Cells in ITP Patients

**Figure 3:**
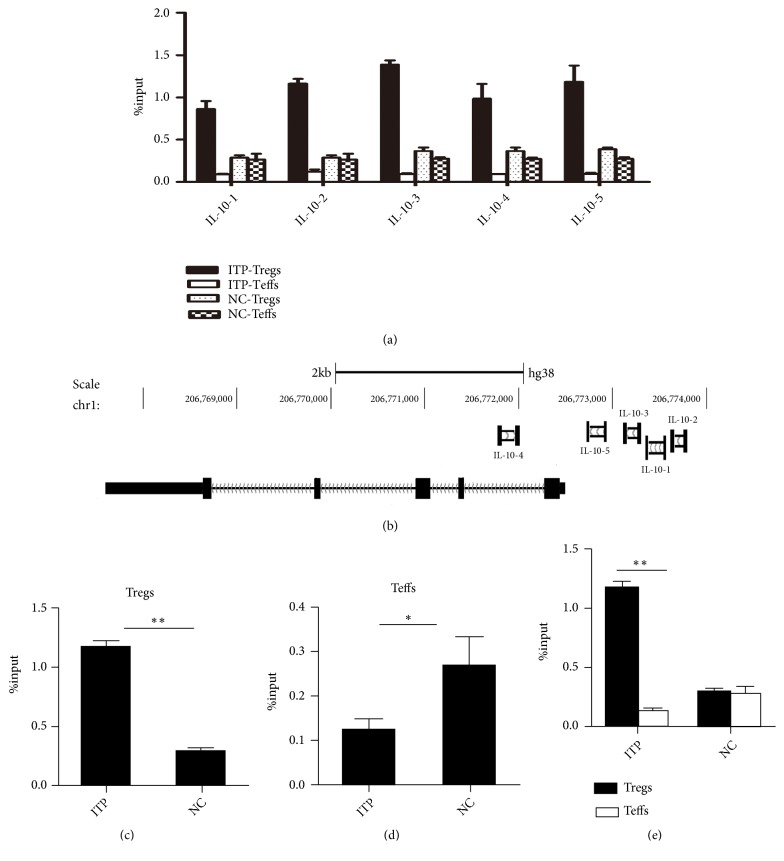
*The target gene IL-10 of IRF4 was associated with the inhibition function of Treg cells in ITP patients.* (a) IRF4 binding used by different primers of IL-10 promoter of CD4^+^CD25^hi^Foxp3^+^Treg cells, and CD4^+^CD25^−^Teff cells in ITP and NC groups. (b) The specific location of IRF4 binding to 5 different IL-10 primers. (c) The intensity of IRF4 binding to IL-10 DNA on Tregs in ITP and NC groups. (d) The intensity of IRF4 binding to IL-10 DNA on Teffs in ITP and NC groups. (e) The intensity of IRF4 binding to IL-10 DNA on Tregs and Teffs in ITP and NC groups.* NC:* normal control,* ITP:* ITP group. *∗p* < 0. 05; *∗∗p* < 0. 01.

The expression of IL-10 mRNA, which is IRF4' downstream gene, was measured by ChIP-qPCR (Figures 3(a) and 3(b); Supplementary Table 1). The intensity of IRF4 binding to IL-10 DNA of CD4^+^CD25^hi^Foxp3^+^Treg cells in patients with ITP was higher than that of normal control group (1.16 ± 0.06* vs* 0.29 ± 0.03,* p*=0.0051) Figure 3(c). For CD4^+^CD25^−^Teffs cells, the intensity of IRF4 binding to IL-10 DNA in patients was lower than that in the normal control group (0.09 ± 0.00* vs *0.27 ± 0.01,* p*=0.006), Figure 3(d). While the intensity of IRF4 binding to IL-10 DNA of CD4^+^CD25^hi^Foxp3^+^Tregs was higher than that of CD4^+^CD25^−^Teffs cells in ITP patients (1.16 ± 0.06* vs *0.12 ± 0.03,* p*=0.034). There was no difference between CD4^+^CD25^hi^Foxp3^+^Tregs with CD4^+^CD25^−^Teffs cells in NC group (0.29 ± 0.03 vs 0.27 ± 0.07,* p*=0.804) Figure 3(e).

### 3.4. After RNA Silencing of IRF4 Gene of Treg Cells of Healthy Controls, the Regulation Function of Tregs Was Compromised

**Figure 4:**
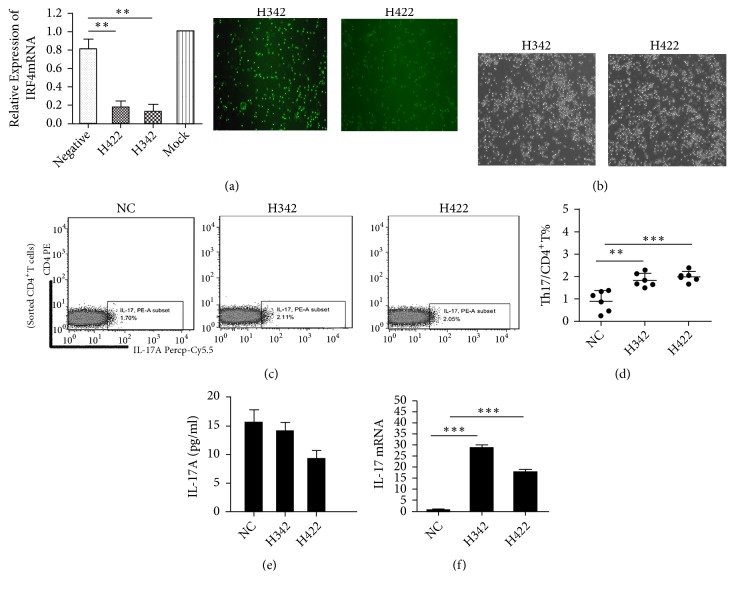
*After RNA silencing of IRF4 gene of Treg cells of healthy controls, the regulation function of Tregs damaged.* (a) Real-time PCR was used to detect the effectiveness of IRF4mRNA silencing.* Negative*: negative control.* Mock: *the calibration set. Representative figure of cells micrographs (×200) of two fragments (H342 and H422) after RNA silencing of IRF4 gene of Tregs. (b) Representative figure of cell micrographs (×200) of two fragments (H342 and H422) after RNA silencing of Tregs, cocultured with CD4^+^CD25^−^Teffs to a ratio of 1:8. (c) Representative dot plots of Th17 cells in NC, H342, and H422 groups after RNA silencing of Tregs, cocultured with CD4^+^CD25^−^T cells. (All CD4^+^T cells were sorted with microbeads.) (d) The percentage of Th17 cells in CD4^+^T cells of three cocultured groups. (e) IL-17 ELISA test for three groups' coculture supernatant. (f) The expression of IL-17mRNA of three groups after being cocultured.* H342: *IRF4-Homo-342,* H422:* IRF4-Homo-422;* NC:* normal control. *∗p* < 0. 05; *∗∗p* < 0. 01; *∗∗∗p* < 0. 001.

IRF4 RNA silencing of CD4^+^CD25^hi^Foxp3^+^Treg cells from healthy control group (Figure 4(a)): Teffs were cultured with Tregs at 8:1 ratio or without Tregs (Figure 4(b)). Then the secretion of extracellular IL-17 cytokines of Th17 cells was measured. The data indicated that the regulation function of Tregs was damaged after the IRF4 gene silencing. The percentage of Th17 cells in CD4^+^ T cells showed a significant high level when compared with the control group after cocultured with CD4^+^CD25^−^T cells (H324 (1.84 ± 0.12) %* vs* (0.91 ± 0.19) %,* p=*0.002; H422 (2.00 ± 0.10) %* vs* (0.91 ± 0.19) %,* p*=0.005), Figures 4(c) and 4(d). But the secretion of extracellular IL-17 had no change compared before and after coculture (H342 13.99 ± 1.572 pg/ml* vs* 15.49 ± 2.29 pg/ml,* p*=0.6047; H422 9.13 ± 1.58 pg/ml* vs* 15.49 ± 2.29 pg/ml,* p*=0.0516), Figure 4(e). The expression of IL-17mRNA was measured after Treg cells were interfered and cocultured with CD4^+^CD25^−^Teffs. The data showed that IL-17mRNA was increased after coculture (H342 28.57 ± 1.51* vs* 0.43 ± 0.30,* p*<0.0001; H422 17.60 ± 1.37* vs* 0.43 ± 0.30,* p*=0.0003), Figure 4(f).

### 3.5. Abnormal Metabolism of Treg and Teff Cells in ITP Patients

**Figure 5:**
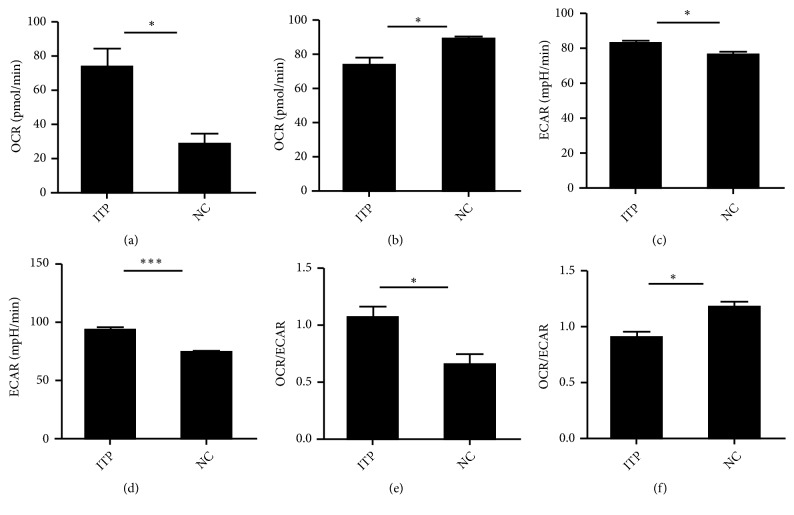
*Abnormal metabolism of Treg and Teff cells in ITP patients.* (a) The oxygen consumption rate (OCR) of Treg cells in ITP and NC groups. (b) OCR of Teff cells in ITP and NC groups. (c) The extracellular media acidification rate (ECAR) of Teff cells in ITP and NC groups. (d) ECAR of Treg cells in ITP and NC groups. (e) The ratio of oxygen consumption to lactate production (OCR/ECAR) of Tregs in ITP and NC groups. (f) The ratio of OCR/ECAR of Teff cells in ITP and NC groups. *∗p* < 0. 05; *∗∗p* < 0. 01; *∗∗∗p* < 0. 001.

The role of Tregs and Teffs metabolism was assessed in ITP patients to explore the Tregs and Teffs experience metabolic reprogramming to support proliferation and immunological function [11]. The oxygen consumption rate (OCR) of Treg cells in ITP patients was higher than that of the control group (73.03 ± 11.32* vs* 27.97 ± 6.46,* p*=0.0135), Figure 5(a). For the Teffs, lower level OCR was found in ITP group compared to NC group (70.58 ± 8.65* vs* 89.25 ± 1.37,* p*=0.0171), Figure 5(b). The extracellular media acidification rate (ECAR) of Teff cells was higher than that of the controls (82.23 ± 1.15* vs* 75.90 ± 1.55,* p*=0.0671), Figure 5(c). ECAR of Tregs showed a higher level compared with NC group (93.20 ± 2.27* vs* 72.98 ± 0.35,* p*=0.0001) and compared with Teffs (93.20 ± 2.27* vs *82.04 ± 1.47,* p*=0.0061), Figure 5(d). The ratio of oxygen consumption to lactate production (OCR/ECAR) of Tregs in patients with ITP increased compared with NC group (1.06 ± 0.10* vs* 0.66 ± 0.09,* p*=0.0224), Figure 5(e). The ratio of OCR/ECAR of Teffs was decreased in ITP patients compared with NC group (0.90 ± 0.054* vs* 1.18 ± 0.04,* p*=0.0031), Figure 5(f).

## 4. Discussion

ITP is known as an autoimmune disease with decreased Treg cells at its onset. Our previous study has indicated that the balance of Th17/Treg is skewed towards Th17 cells, and Th17/Treg ratio might be associated with the clinical diversity of ITP patients at disease onset [11]. The current study found that the expression levels of IRF4 gene and protein of Treg cells in patients with ITP were lower than that of healthy volunteers. The RNA interference targeting IRF4 genes in Treg cells can weaken Treg cells' inhibition of Th17 cells. Furthermore, IRF4-deficient Treg cells showed compromised immunosuppressive function and impaired suppressive activity in ITP patients.

IL-10 is a vital cytokine in immune regulation. It has been shown that Treg cells from IL-10-/- mice lose the ability to inhibit Th17 cells [41]. Such that Treg cells' inhibition function to Th17 cells depends on IL-10 in mice [27]. IL-10 secretion of Tregs was found to be decreased in ITP patients [11, 22]. Insufficient secretion of IL-10 causes impairment of inhibitory capability of Tregs against Teffs in newly diagnosed ITP patients [22].

Although not all but 5 samples for each group were examined due to the sparse of the blood specimens, the expression level of IRF4mRNA and IRF4 protein of Treg cells in ITP patients did show to be lower than that of healthy volunteers, indicating that the immunosuppressive function of IRF4-deficient Treg cells was impaired in ITP setting. To IRF4 protein, the differences in protein images were not obvious, but statistically significant. Several studies have shown that IRF4 is a main regulator connecting with the differentiation and function of Treg and Th17 cells [42–46]. The suppressive activity of Treg cells in ITP patients was also found to be impaired [4]. Evidence showed that the expression of the transcription factor Blimp-1 defined a population of Treg cells that localized mainly to mucosal sites and produced IL-10. The transcription factor IRF4 was indispensable for Blimp-1 expression and for the differentiation of Treg cells [47, 48]. Chromatin immunoprecipitation followed by qPCR analysis (ChIP-qPCR) is a widely used technique to study gene expression. In the current study, the target gene IL-10 of IRF4 on CD4^+^CD25^hi^Foxp3^+^Treg cells was studied. The results showed that the intensity of IRF4 binding to IL-10 DNA on Tregs was higher in patients with ITP than that of normal controls and CD4^+^CD25^−^Teffs. Conversely, the intensity of IRF4 binding to IL-10 DNA on CD4^+^CD25^−^Teffs was lower than normal control group. No significant difference was found between Tregs and Teffs in healthy volunteers. Our data also showed that the secretion of IL-10 of Treg cells in ITP patients was lower than that of healthy controls. In addition, abnormal expression of interleukin regulatory factor 4 gene and abnormal metabolism in Treg cells were detected in ITP patients. These data suggested that IL-10 secretion of Tregs was damaged in ITP patients, although there is enhanced binding force of IRF4 with IL-10 gene which should be compensatory at the onset of the disease. The genetic abnormality of IRF4 on Treg cells might affect the immunosuppression function of Treg cells directly. IRF4 dysfunction of Treg cells results in Th17/Treg imbalances and also may be associated with the secretion of IL-10 through the regulation of metabolism of Tregs.

RNA silencing of IRF4 gene of Treg cells in healthy controls led to the impairment of the regulation function of Tregs to Th17 cells. After IRF4 gene silencing, the ratio of Th17/Treg was increased, likewise as the impaired IRF4 gene of Tregs at onset of ITP.

The percentage of Th17 cells in CD4^+^ T cells showed a higher level compared with the control group after cocultured with CD4^+^CD25^−^Teff cells. The effect of Tregs' suppression on Teffs was improved when IL-10 was added to the coculture system. The percentages of Th17 and IL-17 increased in the coculture system after IL-10 was added. The data showed that IRF4 has high affinity with IL-10 DNA on Treg cells in ITP. IRF4 is closely related to the Treg cells for their immunosuppressive function. IRF4 expressed in various hematopoietic cells, including B cells, T cells, macrophage and dendritic cell subsets [49–52], plays vital role for T cells differentiation [13, 15, 46, 53]. Our finding suggested that the immunosuppressive function of IRF4-deficient Treg cells was compromised in ITP patients. Consequently the function of Treg cells which is determined by dysfunctional IRF4 regulated IL-10 was also impaired.

The increased glycolysis and decreased oxidative phosphorylation of Teff cells were detected in ITP patients, which indicated that Teff cells in ITP patients were overactivated. It has been shown that IRF4 controls the expression of proteins involved in central metabolic pathways such as glucose uptake, glycolysis, and oxidative phosphorylation and thereby promotes changes in cell metabolism necessary for T cell proliferation and effector functions [54]. Teff cells are dependent on the glucose transporter Glut1 and aerobic glycolysis for proliferation and inflammatory functions, as inhibition of glycolysis or deletion of Glut1 impairs Teff cell function in vivo [35, 36]. Due to the requirement of Seahorse testing technology, the specimens obtained from ITP patients cannot match the requirement for test of OCR and ECAR after IRF4 silencing. Recently, there is study showed that the mTOR-IRF4 may influence the function and activation of peripheral Treg cells [55]. And our data also suggested that there is a link between IRF4 and the metabolism of Treg cells. Treg cell function was impaired, and IL-10 secretion disorder might be related to T cell energy metabolism, which is worthy of further study.

Genetic abnormality of IRF4 on CD4^+^CD25^hi^Foxp3^+^Tregs leads to the dysfunction of Treg cells. CD4^+^CD25^−^effector T cells and CD4^+^CD25^hi^Foxp3^+^Treg cells support their proliferation and immunological function in metabolic reprogramming [31]. The expression of Foxp3 of Tregs results in decreased glucose uptake, glycolysis, and lactate production [56].

The increased glycolysis and high ratio of OCR/ECAR of Tregs were found in ITP patients. IL-17 within the Th17 cells was increased; however there was no change of the expression of extracellular IL-17 cytokines after IRF4 gene silencing. The intensity of IRF4 binding to IL-10 DNA on Tregs was increased in ITP patients. The dysfunction of IL-10 secretion of Tregs resulted in inadequate peripheral IL-10 cytokines. These results highly suggested that the immune function of Treg cells in ITP patients could be related to the changes of cell metabolism as abnormal metabolism of Treg and Teff cells in ITP patients were detected. Treg cells are known to have high amounts of IRF4 that depends on Foxp3 expression. The abnormal expression of Foxp3 could result in IRF4 missing, resulting in unable to start the transcription of downstream gene and compromise the immunosuppressive function of Treg cells in patients with autoimmune diseases [18].

In present study, the abnormal metabolism of Tregs and inadequate secretion of IL-10 seemed to be caused by dysfunction of IRF4 in patients with ITP. The activation of Teff cells was results of abnormal cell metabolism. Taken together, the abnormal secretion of IL-10 and IL-17 was related to the abnormal metabolism of T cells. Further study is needed to validate this mechanism.

## 5. Conclusion

IRF4 gene dysfunction of Treg cells in ITP patients leads to the compromised immunosuppression function of Treg cells and the excessive activation of CD4^+^CD25^−^Teffs. The decrease of IL-10 and the increase of IL-17 are related to both the overactivation of Teff cells and activated glycolysis of Tregs. Abnormal metabolism of Treg and Teff cells may play an important role in ITP pathogenesis.* Further studies are warranted to* understand the metabolic signal transduction and function of Treg and Teff cells that might be potential target for ITP immune regulation.

## Data Availability

The data used to support the findings of this study are available from the corresponding author upon request.

## Supplementary Materials

Supplementary Materials Table 1 is the list of the Primer Sequences, Table 2 is the list of the clinical characteristics of ITP patients, Table 3 is the list of Normal Controls (sex, age, and platelet count no.), and Supplementary 4 shows the methods of flow cytometry analysis, cell purification and culture, and Chromatin Immunoprecipitation (ChIP) in detail.

## References

[1] Wei Y., Ji X.-B., Wang Y.-W. (2016). High-dose dexamethasone vs prednisone for treatment of adult immune thrombocytopenia: a prospective multicenter randomized trial. *Blood*.

[2] Sakaguchi S., Miyara M., Costantino C. M., Hafler D. A. (2010). FOXP3^+^ regulatory T cells in the human immune system. *Nature Reviews Immunology*.

[3] Schmidt A., Marabita F., Kiani N. A. (2018). Time-resolved transcriptome and proteome landscape of human regulatory T cell (Treg) differentiation reveals novel regulators of FOXP3. *BMC Biology*.

[4] Liu B., Zhao H., Poon M.-C. (2007). Abnormality of CD4(+)CD25(+) regulatory T cells in idiopathic thrombocytopenic purpura. *European Journal of Haematology*.

[5] Solanilla A., Pasquet J. M., Viallard J. F. (2005). Platelet-associated CD154 in immune thrombocytopenic purpura. *Blood*.

[6] Nomura S., Kuwana M., Ikeda Y. (2003). Induction of T-cell tolerance in a patient with idiopathic thrombocytopenic purpura by single injection of humanized monoclonal antibody to CD40 ligand. *Autoimmunity*.

[7] Baba N., Rubio M., Sarfati M. (2010). Interplay between CD45RA+ regulatory T cells and TNF-*α* in the regulation of human Th17 differentiation. *International Immunology*.

[8] Nistala K., Wedderburn L. R. (2009). Th17 and regulatory T cells: rebalancing pro- and anti-inflammatory forces in autoimmune arthritis. *Rheumatology*.

[9] Khan M. A., Moeez S., Akhtar S. (2013). T-regulatory cell-mediated immune tolerance as a potential immunotherapeutic strategy to facilitate graft survival. *Blood Transfusion*.

[10] Ma J., Yu J., Tao X., Cai L., Wang J., Zheng S. G. (2010). The imbalance between regulatory and IL-17-secreting CD4+ T cells in lupus patients. *Clinical Rheumatology*.

[11] Ji L., Zhan Y., Hua F. (2012). The ratio of Treg/Th17 cells correlates with the disease activity of primary immune thrombocytopenia. *PLoS ONE*.

[12] Biswas P. S., Bhagat G., Pernis A. B. (2010). IRF4 and its regulators: evolving insights into the pathogenesis of inflammatory arthritis?. *Immunological Reviews*.

[13] Brüstle A., Heink S., Huber M. (2007). The development of inflammatory T(H)-17 cells requires interferon-regulatory factor 4. *Nature Immunology*.

[14] Staudt V., Bothur E., Klein M. (2010). Interferon-regulatory factor 4 is essential for the developmental program of T helper 9 cells. *Immunity*.

[15] Bollig N., Brustle A., Kellner K. (2012). Transcription factor IRF4 determines germinal center formation through follicular T-helper cell differentiation. *Proceedings of the National Acadamy of Sciences of the United States of America*.

[16] Nayar R., Schutten E., Bautista B. (2014). Graded levels of IRF4 regulate CD8+ T cell differentiation and expansion, but not attrition, in response to acute virus infection. *The Journal of Immunology*.

[17] Manzano M., Patil A., Waldrop A., Dave S. S., Behdad A., Gottwein E. (2018). Gene essentiality landscape and druggable oncogenic dependencies in herpesviral primary effusion lymphoma. *Nature Communications*.

[18] Zheng Y., Chaudhry A., Kas A. (2009). Regulatory T-cell suppressor program co-opts transcription factor IRF4 to control T(H)2 responses. *Nature*.

[19] Chen Q., Yang W., Gupta S. (2008). IRF-4-binding protein inhibits interleukin-17 and interleukin-21 production by controlling the activity of IRF-4 transcription factor. *Immunity*.

[20] Sharif K., Watad A., Coplan L. (2018). The role of stress in the mosaic of autoimmunity: An overlooked association. *Autoimmunity Reviews*.

[21] Li M. O., Flavell R. A. (2008). Contextual regulation of inflammation: a duet by transforming growth factor-*β* and interleukin-10. *Immunity*.

[22] Li F., Ji L., Wang W. (2015). Insufficient secretion of IL-10 by Tregs compromised its control on over-activated CD4+ T effector cells in newly diagnosed adult immune thrombocytopenia patients. *Immunologic Research*.

[23] Ding L., Shevach E. M. (1992). IL-10 inhibits mitogen-induced T cell proliferation by selectively inhibiting macrophage costimulatory function. *The Journal of Immunology*.

[24] Fitzgerald D. C., Zhang G. X., El-Behi M. (2007). Suppression of autoimmune inflammation of the central nervous system by interleukin 10 secreted by interleukin 27-stimulated T cells. *Nature Immunology*.

[25] McGeachy M. J., Bak-Jensen K. S., Chen Y. (2007). TGF-beta and IL-6 drive the production of IL-17 and IL-10 by T cells and restrain T(H)-17 cell-mediated pathology. *Nature Immunology*.

[26] Chaudhry A., Samstein R. M., Treuting P. (2011). Interleukin-10 signaling in regulatory T cells is required for suppression of Th17 cell-mediated inflammation. *Immunity*.

[27] Huber S., Gagliani N., Esplugues E. (2011). Th17 cells express interleukin-10 receptor and are controlled by Foxp3(-) and Foxp3+ regulatory CD4+ T cells in an interleukin-10-dependent manner. *Immunity*.

[28] De Kouchkovsky D., Esensten J. H., Rosenthal W. L., Morar M. M., Bluestone J. A., Jeker L. T. (2013). MicroRNA-17-92 regulates IL-10 production by regulatory T cells and control of experimental autoimmune encephalomyelitis. *The Journal of Immunology*.

[29] Kornete M., Sgouroudis E., Piccirillo C. A. (2012). ICOS-dependent homeostasis and function of Foxp3+ regulatory T cells in islets of nonobese diabetic mice. *The Journal of Immunology*.

[30] Moon S.-J., Park J.-S., Heo Y.-J. (2013). In vivo action of IL-27: reciprocal regulation of Th17 and Treg cells in collagen-induced arthritis. *Experimental & Molecular Medicine*.

[31] Buck M. D., O’Sullivan D., Pearce E. L. (2015). T cell metabolism drives immunity. *Journal of Experimental Medicine*.

[32] Michalek R. D., Gerriets V. A., Jacobs S. R. (2011). Cutting edge: distinct glycolytic and lipid oxidative metabolic programs are essential for effector and regulatory CD4+ T cell subsets. *The Journal of Immunology*.

[33] Beier U. H., Angelin A., Akimova T. (2015). Essential role of mitochondrial energy metabolism in Foxp3(+) T-regulatory cell function and allograft survival. *The FASEB Journal*.

[34] Gerriets V. A., Kishton R. J., Nichols A. G. (2015). Metabolic programming and PDHK1 control CD4+ T cell subsets and inflammation. *The Journal of Clinical Investigation*.

[35] Chang C.-H., Curtis J. D., Maggi L. B. (2013). Posttranscriptional control of T cell effector function by aerobic glycolysis. *Cell*.

[36] Macintyre A. N., Gerriets V. A., Nichols A. G. (2014). The glucose transporter Glut1 is selectively essential for CD4 T cell activation and effector function. *Cell Metabolism*.

[37] Sawada L., Nagano Y., Hasegawa A. (2017). IL-10-mediated signals act as a switch for lymphoproliferation in Human T-cell leukemia virus type-1 infection by activating the STAT3 and IRF4 pathways. *PLoS Pathogens*.

[38] Rodeghiero F., Stasi R., Gernsheimer T. (2009). Standardization of terminology, definitions and outcome criteria in immune thrombocytopenic purpura of adults and children: report from an international working group. *Blood*.

[39] Provan D., Stasi R., Newland A. C. (2010). International consensus report on the investigation and management of primary immune thrombocytopenia. *Blood*.

[40] Pelletier M., Billingham L. K., Ramaswamy M., Siegel R. M. (2014). Extracellular flux analysis to monitor glycolytic rates and mitochondrial oxygen consumption. *Methods in Enzymology*.

[41] Kochetkova I., Golden S., Holderness K., Callis G., Pascual D. W. (2010). IL-35 stimulation of CD39^+^ regulatory T cells confers protection against collagen II-induced arthritis via the production of IL-10. *The Journal of Immunology*.

[42] Valdez P. A., Vithayathil P. J., Janelsins B. M., Shaffer A. L., Williamson P. R., Datta S. K. (2012). Prostaglandin E2 suppresses antifungal immunity by inhibiting interferon regulatory factor 4 function and interleukin-17 expression in T cells. *Immunity*.

[43] Flutter B., Nestle F. O. (2013). What on "Irf" Is this gene 4? Irf4 transcription-factor-dependent dendritic cells are required for T helper 2 cell responses in murine skin. *Immunity*.

[44] Krishnamoorthy V., Kannanganat S., Maienschein-Cline M. (2017). The IRF4 Gene Regulatory Module Functions as a Read-Write Integrator to Dynamically Coordinate T Helper Cell Fate. *Immunity*.

[45] Vasanthakumar A., Moro K., Xin A. (2015). The transcriptional regulators IRF4, BATF and IL-33 orchestrate development and maintenance of adipose tissue–resident regulatory T cells. *Nature Immunology*.

[46] Myers D. R., Lau T., Markegard E. (2017). Tonic LAT-HDAC7 signals sustain Nur77 and Irf4 expression to tune naive CD4 T cells. *Cell Reports*.

[47] Cretney E., Xin A., Shi W. (2011). The transcription factors Blimp-1 and IRF4 jointly control the differentiation and function of effector regulatory T cells. *Nature Immunology*.

[48] Johnston R. J., Poholek A. C., DiToro D. (2009). Bcl6 and Blimp-1 are reciprocal and antagonistic regulators of T follicular helper cell differentiation. *Science*.

[49] Huber M., Lohoff M. (2014). IRF4 at the crossroads of effector T-cell fate decision. *European Journal of Immunology*.

[50] Suzuki S., Honma K., Matsuyama T. (2004). Critical roles of interferon regulatory factor 4 in CD11bhighCD8alpha- dendritic cell development. *Proceedings of the National Academy of Sciences of the United States of America*.

[51] Lugt B. V., Khan A. A., Hackney J. A. (2014). Transcriptional programming of dendritic cells for enhanced MHC class II antigen presentation. *Nature Immunology*.

[52] Bajaña S., Roach K., Turner S., Paul J., Kovats S. (2012). IRF4 promotes cutaneous dendritic cell migration to lymph nodes during homeostasis and inflammation. *The Journal of Immunology*.

[53] Huber M., Brüstle A., Reinhard K. (2008). IRF4 is essential for IL-21-mediated induction, amplification, and stabilization of the Th17 phenotype. *Proceedings of the National Acadamy of Sciences of the United States of America*.

[54] Man K., Miasari M., Shi W. (2013). The transcription factor IRF4 is essential for TCR affinity–mediated metabolic programming and clonal expansion of T cells. *Nature Immunology*.

[55] Chapman N. M., Zeng H., Nguyen T. M. (2018). mTOR coordinates transcriptional programs and mitochondrial metabolism of activated Treg subsets to protect tissue homeostasis. *Nature Communications*.

[56] Gerriets V. A., Kishton R. J., Johnson M. O. (2016). Foxp3 and Toll-like receptor signaling balance Treg cell anabolic metabolism for suppression. *Nature Immunology*.

