# Supplementary material for: Transcription Factor IRF4 Dysfunction Affects the Immunosuppressive Function of Treg Cells in Patients with Primary Immune Thrombocytopenia
Source: Biomed Res Int. 2019 Jul 10;2019:1050285. doi: 10.1155/2019/1050285 (PMC6652070; doi:10.1155/2019/1050285)
Supplement: Supplementary Materials — Table 1 is the list of the Primer Sequences, Table 2 is the list of the clinical characteristics of ITP patients, Table 3 is the list of Normal Controls (sex, age, and platelet count no.), and Supplementary 4 shows the methods of flow cytometry analysis, cell purification and culture, and Chromatin Immunoprecipitation (ChIP) in detail. [file 1050285.f1.docx]

| **Table1** The Primer Sequences | |
| --- | --- |
| **gene** | **Primer Sequence** |
| IL-17Human | F:5’ GTCAACCTGACATCCATAACC3’ |
| IRF4 | F:5’ CAGTTTCACCGCTCGATCTT3’ |
| IL-10-1 | F:5’ GTAGGGATAGGTAAGAGGAAAGTA3’ |
|  | R:5’ TCTCCAGCACATAGAATGAAAC3’ |
| IL-10-2 | F:5’ AGGTGAAGGCTCAATCAAAGGA3’ |
|  | R:5’ GGCGAGGAGTGTTGCTCTACC3’ |
| IL-10-3 | F:5’ TTCTATGTGCTGGAGATGGTG3’ |
|  | R:5’ TTTATAGTGAGCAAACTGAGGC3’ |
| IL-10-4 | F:5’ AGAAATGTCTGATTCGAGGTG3’ |
|  | R:5’ TGTAGAGTGCTTCCCTAAACC3’ |
| IL-10-5 | F:5’ AAGGAGAAGTCTTGGGTATTCATC3’ |
|  | R:5’ GCTGTGGGTTCTCATTCGC3’ |

| **Table2** Clinical characteristics of ITP patients | | | |
| --- | --- | --- | --- |
| Patient no. | Sex | Age(years) | Platelet counts (×10^9^/l) |
| ITP1 | F | 56 | 26 |
| ITP2 | F | 77 | 22 |
| ITP3 | M | 51 | 27 |
| ITP4 | F | 77 | 11 |
| ITP5 | M | 68 | 29 |
| ITP6 | F | 67 | 9 |
| ITP7 | F | 66 | 21 |
| ITP8 | M | 64 | 1 |
| ITP9 | F | 68 | 21 |
| ITP10 | M | 71 | 27 |
| ITP11 | M | 56 | 31 |
| ITP12 | F | 77 | 19 |
| ITP13 | M | 54 | 11 |
| ITP14 | F | 66 | 27 |
| ITP15 | M | 67 | 11 |
| ITP16 | M | 72 | 21 |
| ITP17 | F | 68 | 29 |
| ITP18 | F | 56 | 29 |
| ITP19 | F | 57 | 29 |
| ITP20 | M | 42 | 23 |
| ITP21 | M | 59 | 16 |
| ITP22 | F | 76 | 8 |
| ITP23 | M | 33 | 6 |
| ITP24 | F | 42 | 23 |
| ITP25 | M | 19 | 3 |
| ITP26 | F | 55 | 6 |
| ITP27 | F | 73 | 18 |
| ITP28 | F | 56 | 24 |
| ITP29 | M | 24 | 29 |
| ITP30 | M | 77 | 22 |
| ITP31 | F | 61 | 30 |
| ITP32 | F | 58 | 30 |
| ITP33 | M | 31 | 41 |
| ITP34 | F | 43 | 20 |
| ITP35 | F | 70 | 37 |
| ITP36 | M | 36 | 39 |
|  | M:F = 16:20 | 49(19-77) | 22(1-39) |

| **Table3** Normol Controls | | | |
| --- | --- | --- | --- |
| Normol Controls no.(NC) | Sex | Age(years) | Platelet counts (×10^9^/l) |
| NC1 | F | 52 | 225 |
| NC2 | F | 55 | 176 |
| NC3 | M | 24 | 176 |
| NC4 | F | 56 | 129 |
| NC5 | F | 70 | 214 |
| NC6 | F | 43 | 161 |
| NC7 | M | 29 | 202 |
| NC8 | F | 47 | 151 |
| NC9 | F | 46 | 202 |
| NC10 | F | 57 | 188 |
| NC11 | F | 68 | 172 |
| NC12 | F | 49 | 232 |
| NC13 | F | 52 | 162 |
| NC14 | F | 58 | 155 |
| NC15 | F | 61 | 198 |
| NC16 | M | 67 | 184 |
| NC17 | F | 72 | 201 |
| NC18 | M | 31 | 173 |
| NC19 | M | 36 | 164 |
| NC20 | F | 70 | 189 |
|  | M:F=5:15 | 54(24-72) | 180(129-232) |

**Supplementary 4**

**Flow cytometry analysis**
To test CD4^+^CD25^hi^Foxp3^+^Treg cells, 1 × 10^6^ PBMCs were stained with CD4 FITC (eBioscience, San Diego, California, USA, Cat# 11-0048-42), CD25 PE-CY7 (BD Bioscience, Cat# 506225). After incubation at 4℃ for 20 min with Foxp3 Fixation/Permeabilization Buffer (eBioscience, Cat# 00-5521), cells were washed twice with Permeabilization Buffer (eBioscience, Cat# 00-8333) and stained with Foxp3 V450 (BD Bioscience, Cat# 560459). To detect Th17 cells, 1 × 10^6^ PBMCs were adjusted concentration as 5 × 10^5^/ml in RPMI1640 medium supplemented with 10% heat-inactivated fetal bovine serum, 2 mM L-glutamine, 200 U/ml penicillin, and 100 μg/ml streptomycin, and cultured in 24-well plates overnight. Before stimulation, the supernatants were collected for IL-17 ELISA test. The concentration of PBMCs were adjusted as 5 × 10^5^/ml in RPMI1640 medium supplemented with 10% heat-inactivated fetal bovine serum, 2 mM L-glutamine, 200 U/ml penicillin, and 100 μg/ml streptomycin, and stimulated with 50 ng/ml phorbol myristate acetate (PMA, Sigma-Aldrich, St. Louis, Missouri, USA, Cat# P8139) and 500 ng/ml ionomycin (Sigma-Aldrich, Cat# I9657) for 4 hours. End in 2 hours after incubation, 1 ul/ml brefeldin A solution (BFA, Biolegend, Cat# 420602) was added into the culture system. Then PBMCs were stained with CD4 FITC, CD25 PE-CY7. After the fixation and permeabilization step described above, the cells were stained with IL-17A PerCP-Cy5.5 (BD Bioscience, Cat# 560799). For the co-culture system after transfection experiment, the cells were stimulated by PMA, ionomycin, and BFA with the same condition of PBMCs mentioned above. Cells were stained with IL-17A PerCP-Cy5.5. All steps according to the manufacturer’s protocol. Acquisition was performed on a FACS Aria Ⅱ flow cytometer (BD biosciences, USA) and then analyzed using Flowjo software version 7.6.1(Tritar Inc., San Carlos, California, USA).

**Cell purification and culture**

Dead cells were removed by dead cell removal kit (Miltenyi Biotec, Auburn, California, USA, Cat# 130-090-101). CD4^+^CD25^hi^Foxp3^+^Treg cells and CD4^+^CD25^-^Teffs were isolated from PBMC using the CD4^+^CD25^+^Treg cells isolation kit (Miltenyi Biotec, Cat# 130-091-301) according to manufacturer’s instruction. CD4^+^ T cells were purified by negative selection and then incubated with CD25 microbeads followed by separation by using a LD column. The negative fraction CD4^+^CD25^-^Teffs was collected, and the positive fraction was passed over a MS column to enrich in Treg cells. The purification of Tregs and Teffs were determined by FACS Aria II flow cytometer (BD biosciences, San Jose, California, USA) using CD4 APC (Biolegend, San Diego, California, USA, Cat# 300514) and CD25 PE (Biolegend, Cat# 302605).

Before culture, 24-well round-bottom plates were pre-incubated with anti-CD3 (10 μg/ml; Biolegend, Cat# 300414) at 37℃ for 2 hours. Teffs and Tregs were adjusted concentration at 1×10^6^/ml in RPMI1640 medium supplemented with 10% heat-inactivated fetal bovine serum, 2 mM L-glutamine, 200 U/ml penicillin, and 100 ug/ml streptomycin. Teffs were cultured with Tregs at 8:1 ratio or without Tregs. The cells were cultured in 1 ml per well in 24-well round-bottom plates which were pre-incubated and stimulated with anti-CD28 (2.5 μg/ml, Biolegend, Cat# 302914) and 10 ng/ml interleukin-2 (IL-2; Biolegend, Cat# 589102).

**Chromatin Immunoprecipitation (ChIP)**

CD4^+^CD25^+^Treg cells and CD4^+^CD25^-^Teffs were separated from PBMCs of ITP patients and healthy volunteers with magnetic bead and transferred to the centrifuge. Formaldehyde (270 μl, 37%) was added and their final concentration was adjusted 1% for 10 min incubation at room temperature. Then 125 mM ice-cold glycine (505 ul 2.5 M) was added for another 5 min at room temperature. Cells were washed with iced phosphate buffered saline (PBS) three times at 135g, 4℃. One milliliter PBS with protease inhibitors mixture was added then centrifuged for 5 min at 800g, 4°C. Sand supernatant was removed carefully. Protease inhibitors (final concentration of 1 x) were added to lysis buffer 1 (50 mM Hepes-KOH pH 7.5; NaCl 140 mM; EDTA 1 mM; glycerol 10%; NP-40 0.5%; Tritonx-100 0.25%) and lysis buffer 2 (10 mM Tris-HCl pH 8.0; NaCl 100 mM; EDTA 1 mM pH 8.0; Na-Deoxycholate 0.1% Protease inhibitors). Pellets were resuspended in 1mL lysis buffer 1 and swirled after 10 min at 4℃, then centrifuged for 5 min at 800g, 4°C and abandoned the supernatant. The pellets were resuspended in 300 μL of lysis buffer 2 and placed on the ice for 30 min. Then the pellets sonicated on the ice with a Bioruptor (Diagenode, 10 pulses of 15 seconds each at 15% amplitude with 30 seconds off between each pulse). The generated fragments were approximately 500 bp long, as determined experimentally by Agileng 2100 software. 750 μl IP Buffer, 100 μl 5% BSA, Magnetic beads-antibody complex (Sheep anti-Mouse IgG Beads (Invitrogen) and 4 μg anti-IRF4 Ab (Cell Signaling Technology, Cat# 4964s) 30 μl were added, 0.6 μl Salmon Sperm DNA, 100 μl fragments of chromosome, 10 μl protease inhibitors, PMSF 10 μl were mixed well. After incubation at 4°C overnight, the beads were subsequently washed with buffer A, (0.1% SDS, 1% Triton X-100, 2 mM EDTA, 20 mM Tris-HCl (pH 8.0), 0.15 M NaCl), buffer B, (0.1% SDS, 1% Triton X-100, 2 mM EDTA, 20 mM Tris-HCl (pH 8.0), 0.5 M NaCl) and buffer C(according to the instructions) (0.25 M LiCl, 1% NP40, 1% Na-deoxycholate, 1 mM EDTA, Tris-HCl (pH 8.0), at 4°C with permanent rotation for 10 mins. Following two washes with TE buffer (10 mM Tris-HCl, 10 mM EDTA, pH 8.0), samples were eluted in 150 μl Chip Elution Buffer for 1 hour at 65°C. Samples were then added 8 μl proteinase K and 6 μl NaCI (5 M) and cross-links reversed at 65°C overnight. DNA was purified using QIAquick PCR Purification Kit (QIAGEN).
